# Supplementary material for: Limits of aerobic metabolism in cancer cells
Source: Sci Rep. 2017 Oct 18;7:13488. doi: 10.1038/s41598-017-14071-y (PMC5647437; doi:10.1038/s41598-017-14071-y)
Supplement: Supplementary file 1 — Supplementary Information [file 41598_2017_14071_MOESM1_ESM.pdf]

Supplementary Information

Limits of aerobic metabolism in cancer cells

Jorge Fernandez-de-Cossio-Diaz[1] and Alexei Vazquez[2,3]*

[1]Center of Molecular Immunology, Havana, Cuba

[2]Cancer Research UK Beatson Institute, Glasgow, UK

[3]Institute for Cancer Sciences, University of Glasgow, Glasgow, UK

Component	% of cell weight	% of cell dry weight				Energy demand (mol ATP/L cell)		
Water	70		Unit	Unit MW (g/mol)	Unit concentration (M)	Per precursor	From glucose	NADH OxPhos (mol ATP/L cell)
Ref.	[1]			[2]		[2]		
Protein	18	60.0	Amino acids	109	1.10	4.30	4.62	1.57
Lipids	5	16.7	Ac	28	1.19	0.88	1.04	5.95
Polysaccharides	2	6.7	Glucose	180	0.07	0.00	0.15	0.00
RNA	1.1	3.7	Ribonucl.	331	0.02	0.40	0.06	0.06
DNA	0.25	0.8	Deoxyribonucl.	332	0.01	1.37	0.02	0.01
Total	96.4	87.8					6.0	7.6
Synthesis from glucose: (+) produced, (-) consumed								
Molecule	Frequency in precursor	ATP/molecule	ATP/precursor	NADH	glu -> akg	Net NADH produced per precursor unit		
Ref.	[2]	[3]		[3]	[3]			
ala	0.087	1	0.087	1	1	0		
arg	0.055	0	0	2	1	0.055		
asn	0.042	-1	-0.042	1	1	0		
asp	0.052	0	0	1	1	0		
cys	0.021	0	0	2	1	0.021		
glu	0.056	1	0.056	3	0	0.168		
gln	0.047	0	0	3	0	0.141		
gly	0.078	0	0	2	1	0.078		
pro	0.045	0	0	1	0	0.045		
ser	0.062	0	0	2	1	0.062		
amino acids	0.545		0.101			0.570		
AcCoA	1.00	0	0	2	0	2		
polysaccharides	1.00	-2	-2	0	0	0		
purine	0.50	-6	-3	1	0	1		
pyrimidine	0.50	-3	-1.5	1	0	1		
RNA			-2.25			1		
DNA			-2.25			1		
Parameter	Value	Units	Ref.					
Maintenace energy	17	pmol/cell/day	[4]					
Cell volume	3.4	pL	[4]					
Maintenance energy	5	M/h						
Cell specific volume	0.005	L/g	[5]					

Table S1: Biosynthetic demands of cell biomass duplication from glucose.

		r_M		
Cell line/tissue	Cell type	mmol ATP/g mitochondria protein/min	mol ATP/L mitochondria/min	Reference (PMID)
PC-3	prostate cancer	0.11	0.042	[6]
HeLa	cervical cancer	0.13	0.049	[7]
Liver	normal liver	0.18	0.068	[8]
Liver	normal liver	0.25	0.095	[9]
Lateralis	skeletal muscle	0.35	0.133	[10]
Heart	heart muscle	0.40	0.152	[11]
Gastrocnemius medialis	skeletal muscle	0.52	0.198	[12]
Soleus	skeletal muscle	0.70	0.266	[8]
Yeast	microorganism	0.75	0.285	[13]
Heart	heart muscle	0.84	0.319	[8]
Plantaris	skeletal muscle	1.00	0.380	[8]
Glycolysis	muscle	1.20	1.519	[14]
	Specific volume			
Mitochondria specific volume	2.63	mL/g		[15]
Globular proteins specific volume	0.79	mL/g		[16]

Table S2: ATP generating capacity of mitochondria from different tissues and cells lines and from glycolysis.

Reference (PMID)	Cell line	Td (h)	Td Reference (PMID)	ATP production/demand (mol ATP/L/h)		
				Total	Growth	Maintenance
[17]	HeLa	25	[17]	0.55	0.17	0.39
[18]	HCT116	17.4	NCI60	0.40	0.24	0.16
	HT29	19.5	NCI60	0.31	0.21	0.09
[19]	TOV21G	25	[20]	0.73	0.17	0.57
	ES2	19	[20]	0.73	0.22	0.51
	OVCAR3	51	[20]	0.45	0.08	0.37
	DOV13	21.3	[21]	0.43	0.20	0.23
	OVCA433	29.4	[21]	0.52	0.14	0.37
	OVCA429	29.4	[21]	0.49	0.14	0.35
	OVCA420	25.6	[22]	0.34	0.16	0.18
	Average					5.4
	STD					2.6

Table S3: Cell energy maintenance of cancer cells.

Cell line	Doubling time	Mitochondrial volume %
LE:SR	28.7	0.71
BR:HS578T	53.8	2.35
LE:MOLT-4	27.9	2.52
BR:MDA-MB-231	41.9	2.67
ME:SK-MEL-5	25.2	2.68
RE:A498	66.8	2.71
LE:HL-60	28.6	2.84
LE:K-562	19.6	2.86
RE:UO-31	41.7	2.87
CNS:SF-539	35.4	2.89
BR:T-47D	45.5	3.23
ME:SK-MEL-2	45.5	3.30
LE:CCRF-CEM	26.7	3.41
OV:OVCAR-8	26.1	3.49
BR:BT-549	53.9	3.53
LE:RPMI-8226	33.5	3.63
OV:SK-OV-3	48.7	3.69
RE:ACHN	27.5	3.89
RE:CAKI-1	39	3.94
ME:UACC-257	38.5	3.98
CO:HCT-116	17.4	4.06
ME:MDA-MB-435	25.8	4.17
OV:OVCAR-8/ADR	34	4.20
CNS:SF-268	33.1	4.29
CNS:SF-295	29.5	4.31
OV:IGROV1	31	4.42
ME:SK-MEL-28	35.1	4.43
CO:COLO205	23.8	4.60
ME:LOXIMVI	20.5	4.62
RE:786-0	22.4	4.69
CNS:SNB-75	62.8	4.81
LC:NCI-H322M	35.3	5.07
RE:SN12C	29.5	5.07
ME:M14	26.3	5.12
OV:OVCAR-5	48.8	5.15
RE:RXF-393	62.9	5.46
RE:TK-10	51.3	5.57
CO:HT29	19.5	5.72
LC:HOP-62	39	5.79
PR:DU-145	32.3	5.94
ME:MALME-3M	46.2	6.07
CNS:U251	23.8	6.20
LC:NCI-H226	61	6.41
ME:UACC-62	31.3	6.82
CO:HCT-15	20.6	6.99
PR:PC-3	27.1	7.13
CO:SW-620	20.4	7.14
CNS:SNB-19	34.6	7.16
CO:HCC-2998	31.5	7.23
LC:EKVX	43.6	7.41
LC:A549/ATCC	22.9	7.77
OV:OVCAR-3	34.7	7.86
LC:HOP-92	79.5	7.92
LC:NCI-H460	17.8	8.33
CO:KM12	23.7	8.36
BR:MCF7	25.4	8.62
LC:NCI-H522	38.2	8.91
LC:NCI-H23	33.4	9.42
OV:OVCAR-4	41.4	10.47

Table S4: Estimated protein content of the NCI60 cell lines.

References

1. Alberts B: **Molecular biology of the cell**, 5th edn. New York: Garland Science; 2008.
2. Sheikh K, Forster J, Nielsen LK: **Modeling hybridoma cell metabolism using a generic genome-scale metabolic model of Mus musculus**. *Biotechnol Prog* 2005, **21**(1):112-121.
3. Voet D, Voet JG: **Biochemistry**, 4th edn. Hoboken, N.J.: John Wiley & Sons, Inc.; 2011.
4. Kilburn DG, Lilly MD, Webb FC: **The energetics of mammalian cell growth**. *Journal of cell science* 1969, **4**(3):645-654.
5. Frame KK, Hu WS: **Cell volume measurement as an estimation of mammalian cell biomass**. *Biotechnol Bioeng* 1990, **36**(2):191-197.
6. de Bari L, Moro L, Passarella S: **Prostate cancer cells metabolize d-lactate inside mitochondria via a D-lactate dehydrogenase which is more active and highly expressed than in normal cells**. *Febs Lett* 2013, **587**(5):467-473.
7. Kioka H, Kato H, Fujikawa M, Tsukamoto O, Suzuki T, Imamura H, Nakano A, Higo S, Yamazaki S, Matsuzaki T *et al*: **Evaluation of intramitochondrial ATP levels identifies G0/G1 switch gene 2 as a positive regulator of oxidative phosphorylation**. *Proc Natl Acad Sci U S A* 2014, **111**(1):273-278.
8. Short KR, Nygren J, Barazzoni R, Levine J, Nair KS: **T(3) increases mitochondrial ATP production in oxidative muscle despite increased expression of UCP2 and -3**. *American journal of physiology Endocrinology and metabolism* 2001, **280**(5):E761-769.
9. Chinopoulos C, Konrad C, Kiss G, Metelkin E, Torocsik B, Zhang SF, Starkov AA: **Modulation of F0F1-ATP synthase activity by cyclophilin D regulates matrix adenine nucleotide levels**. *Febs J* 2011, **278**(7):1112-1125.
10. Karakelides H, Irving BA, Short KR, O'Brien P, Nair KS: **Age, obesity, and sex effects on insulin sensitivity and skeletal muscle mitochondrial function**. *Diabetes* 2010, **59**(1):89-97.
11. Yoshioka J, Chutkow WA, Lee S, Kim JB, Yan J, Tian R, Lindsey ML, Feener EP, Seidman CE, Seidman JG *et al*: **Deletion of thioredoxin-interacting protein in mice impairs mitochondrial function but protects the myocardium from ischemia-reperfusion injury**. *J Clin Invest* 2012, **122**(1):267-279.
12. Hou XY, Green S, Askew CD, Barker G, Green A, Walker PJ: **Skeletal muscle mitochondrial ATP production rate and walking performance in peripheral arterial disease**. *Clinical physiology and functional imaging* 2002, **22**(3):226-232.
13. Gonzalvez F, Pariselli F, Dupaigne P, Budihardjo I, Lutter M, Antonsson B, Diolez P, Manon S, Martinou JC, Goubern M *et al*: **tBid interaction with cardiolipin primarily orchestrates mitochondrial dysfunctions and subsequently activates Bax and Bak**. *Cell death and differentiation* 2005, **12**(6):614-626.
14. Vazquez A, Liu J, Zhou Y, Oltvai ZN: **Catabolic efficiency of aerobic glycolysis: The Warburg effect revisited.** *Bmc Syst Biol* 2010, **4**:58.

15. Schwerzmann K, Hoppeler H, Kayar SR, Weibel ER: **Oxidative capacity of muscle and mitochondria: correlation of physiological, biochemical, and morphometric characteristics**. *Proc Natl Acad Sci U S A* 1989, **86**(5):1583-1587.
16. Lee B: **Calculation of volume fluctuation for globular protein models**. *Proc Natl Acad Sci U S A* 1983, **80**(2):622-626.
17. Quiros PM, Prado MA, Zamboni N, D'Amico D, Williams RW, Finley D, Gygi SP, Auwerx J: **Multi-omics analysis identifies ATF4 as a key regulator of the mitochondrial stress response in mammals**. *The Journal of cell biology* 2017, **216**(7):2027-2045.
18. Zaytseva YY, Harris JW, Mitov MI, Kim JT, Butterfield DA, Lee EY, Weiss HL, Gao T, Evers BM: **Increased expression of fatty acid synthase provides a survival advantage to colorectal cancer cells via upregulation of cellular respiration**. *Oncotarget* 2015, **6**(22):18891-18904.
19. Dier U, Shin DH, Hemachandra LP, Uusitalo LM, Hempel N: **Bioenergetic analysis of ovarian cancer cell lines: profiling of histological subtypes and identification of a mitochondria-defective cell line**. *Plos One* 2014, **9**(5):e98479.
20. Beaufort CM, Helmijr JC, Piskorz AM, Hoogstraat M, Ruigrok-Ritstier K, Besselink N, Murtaza M, van IWF, Heine AA, Smid M *et al*: **Ovarian cancer cell line panel (OCCP): clinical importance of in vitro morphological subtypes**. *Plos One* 2014, **9**(9):e103988.
21. Huang RY, Wong MK, Tan TZ, Kuay KT, Ng AH, Chung VY, Chu YS, Matsumura N, Lai HC, Lee YF *et al*: **An EMT spectrum defines an anoikis-resistant and spheroidogenic intermediate mesenchymal state that is sensitive to e-cadherin restoration by a src-kinase inhibitor, saracatinib (AZD0530)**. *Cell death & disease* 2013, **4**:e915.
22. Chan QK, Ngan HY, Ip PP, Liu VW, Xue WC, Cheung AN: **Tumor suppressor effect of follistatin-like 1 in ovarian and endometrial carcinogenesis: a differential expression and functional analysis**. *Carcinogenesis* 2009, **30**(1):114-121.
